# Creating Single-Cell Protein-Producing *Bacillus subtilis* Mutants Using Chemical Mutagen and Amino Acid Inhibitors

**DOI:** 10.1155/sci5/8968295

**Published:** 2024-11-29

**Authors:** Indra Berzina, Martins Kalnins, Zane Geiba, Svetlana Raita, Jelizaveta Palcevska, Taras Mika, Kriss Spalvins

**Affiliations:** Institute of Energy Systems and Environment, Riga Technical University, Azenes Street 12/1, LV 1048, Riga, Latvia

**Keywords:** amino acids, amino acid inhibitors, *Bacillus subtilis*, biomass, herbicides, mutagenesis, proteins, single-cell proteins, waste biomass

## Abstract

Due to population growth and climate changes, there is a rising need for alternative food and protein sources to reduce protein scarcity and the environmental impact of food industries. Single-cell proteins (SCPs) have the potential to partially or fully substitute plant- and animal-derived dietary proteins. *Bacillus subtilis* is an appealing bacterium for SCP production because of its fast growth and ability to obtain high protein and essential amino acid (AA) content in its biomass. It is also capable of utilizing a wide range of substrates. *B. subtilis* attractiveness and efficiency can be further enhanced using mutagenesis. In this study, a novel approach to creating mutant strains with enhanced protein and AA content was experimentally validated. The method is based on the application of AA inhibitors for selective pressure to ensure the growth of mutants with enhanced protein and/or AA synthesis capacity. For AA inhibitors, three herbicides were used: glufosinate–ammonium (GA), L-methionine sulfoximine (MSO), and S-(2-aminoethyl)-L-cysteine (AEC). Initially, AA inhibitor doses for the complete inhibition of wild-type (WT) *B. subtilis* strain were determined. Then, *B. subtilis* was treated with EMS chemical mutagen and created mutants were cultivated on a medium containing inhibitory dose of AA inhibitors. Growing samples were selected, analyzed, and compared. The optimal inhibitory concentrations of herbicides for mutant selection were 0.05–0.4 M for GA, 0.01–0.05 M for MSO, and 0.2 M for AEC. The best-performing mutants were selected when using GA—improvement of 7.1 times higher biomass content, 1.5 times higher protein concentration, 1.2 times higher AA content, and 1.2 times higher essential AA index was achieved in comparison with WT *B. subtilis*. Enhanced mutants were also successfully selected when using MSO and AEC. This study demonstrates the potential of using AA inhibitors for the selection of mutants with improved protein and AA profiles.

## 1. Introduction

Ensuring enough dietary protein is crucial for global food and nutrition security. According to the Sustainable Development Goals Report 2023, 1 in 3 people worldwide struggle with moderate to severe food insecurity, and widespread use of highly processed food continues to plague many nations [1]. Furthermore, problems are exaggerated by inefficiencies in feed production in the livestock and aquaculture industries [2]. Single-cell proteins (SCPs) are a promising alternative, and both industries need to find new sources of protein-rich feeds that contain all the amino acids (AAs) needed for a complete diet [3–5].

There is growing interest in alternative, more environmentally sustainable protein sources. Microbial protein or SCP is one such alternative to conventional protein sources. The SCP has high protein content; it contains vitamins, minerals, nucleic acids, and lipids [6, 7]. It is an attractive feed supplement for animal and human nutrition, considering the many health benefits linked to specific microorganisms and the popularity of probiotic supplementation [8]. SCP production has many advantages; for example, it is more resource efficient than agriculture and livestock production, production is rapid, and waste biomass and industrial by-products can be used as the main feedstock [3, 6, 9]. However, there is still a need to increase the competitiveness of technologies.

Bacteria can utilize various substrates for SCP production, including sugars, starches, organic waste, and even nutrient-rich effluents [6]. Bacteria are selected for SCP production due to their fast growth, high protein content, and ability to utilize various nitrogen sources such as ammonia, urea, and organic nitrogen [6, 7]. *Bacillus subtilis*, among other bacterial species, demonstrates efficient protein production potential, offering AA profiles suitable for animal feeds. *B. subtilis* biomass can have very high protein contents reaching up to 71% [6, 10], containing all essential AA needed for ruminant feed [10]. Despite that, there are only a few studies where *Bacillus spp.* is used for SCP production [10–12]. More often, *Bacillus spp.* is used to ferment substrates to enhance the substrate nutritional content and properties [13–17] or when used in mixed culture fermentation [18–22].

Jang et al. emphasized the importance of selecting appropriate *Bacillus* strains for soybean fermentation to optimize AA profiles and enhance flavor development in fermented soybean products [13]. It was determined that *B. licheniformis* can enhance glutamic acid concentration for umami taste, and *B. subtilis* increases essential AA content [13]. Mok et al. investigated the fermentation of *B. subtilis* using okara, a by-product of soybean processing, as the sole nutrient source [15]. Fermentation produced a functional beverage rich in AA, short-chain fatty acids, phenolic compounds, and antioxidants [15]. The beverage exhibited probiotic properties, with a high concentration of viable *B. subtilis* cells [15]. Similarly, Liu et al. found that the fermentation of peanut meal using *B. subtilis* significantly increased protein content, essential AA, and total AA [14]. The hydrolysis of proteins into smaller peptides during fermentation contributed to improved digestibility and bioavailability of proteins [14]. Yang et al. showed that *Sichuan paocai*, Chinese kimchi, inoculation with *B. subtilis* promoted the growth of various lactic acid bacteria while reducing the number of opportunistic pathogenic bacteria [16]. The inoculation also increased the content of important volatile flavor components, organic acids, and AA, which could further help to increase the formation of the palatable taste and flavor [16]. These findings highlight how *B. subtilis* can produce functional and nutritious food with enhanced properties. This suggests that in SCP technology, the strain growing on food by-products will also be able to create functional feed.

Previous studies involving *B. subtilis* have focused more on the bacteria's use as a probiotic in animal feed. The European Union countries and the United States have prohibited the use of antibiotics in livestock and poultry feed because antibiotic-resistant strains are spreading into the environment and can be transmitted to humans through the food chain [23]. *B. subtilis* enzymes increase digestive capabilities and help assimilate nutrients from animal and plant origin under extreme environmental conditions [24]. It can become a multifunctional probiotic bacteria [24].

Many studies have already been conducted where *B. subtilis* has been used as a probiotic in aquaculture [24–27], poultry [28–31], or pig feed [32, 33]. In *Penaeus monodon* juveniles, fermenting a plant protein mix with *Bacillus spp.* improved growth and feed digestibility, indicating its potential as a fishmeal substitute in shrimp feed [25]. Similarly, in other aquatic species such as *Pangasius hypophthalmus* and *Totoaba macdonaldi*, *B. subtilis* supplementation enhanced growth performance, nutrient utilization, and resistance to pathogens [24, 26, 27]. In terrestrial animals such as broiler chickens, ducks, and pigs, *B. subtilis* supplementation improved carcass traits, meat quality, reproductive performance, nutrient digestibility, gut health, and immune modulation [28, 29, 31–33]. In duck breeding, combining L-threonine and *B. subtilis* supplementation improved eggshell quality, hatchability, and body weights of newly hatched ducklings, indicating how to improve overall productivity in duck farming [30]. This bacteria is known for its application in different biotechnologies for the production of a variety of products, including fine chemicals, biopolymers, and proteins [6, 7, 10, 34–37]. *B. subtilis* can be used as a probiotic and can positively affect the consumer [38–41]. However, it has been asserted that *B. subtilis* does not pose human safety concerns at the acceptable daily intake of 3.7 × 10^9^ CFU/kg body weight/day [8]. The mentioned data indicate the advantageous potential of *B. subtilis* use as a feed supplement, and improving its protein content will increase important economic factors such as the product's value and quality.

One of the ways to increase SCP technology competitiveness in the market would be increasing product protein quality and value. Spalvins et al. proposed that treating the protein-producing strain with a mutagen and cultivating it in a selective medium would help create a new novel strain with increased protein concentration and improved essential AA concentrations [3]. For this reason, AA inhibitors can be used to create a selective medium. Most AA inhibitors that are available are used in agriculture as herbicides, and the majority of research conducted on using these compounds is regarding their practical and cost-effectiveness in weed management [42–45]. Most research on herbicides focuses on their effects on plant biosynthetic pathways, with limited information on their impact on single-cell organisms, which is crucial when choosing AA inhibitors for selecting SCP-producing mutants [3].

In this study, the proposed approach of AA inhibitor-mediated mutant selection was validated. *B. subtilis* was used in experiments to create and select mutant strains with enhanced protein-producing abilities. The technique for improving strains with mutagenesis has been useful for improving the production of enzymes [6, 7, 10, 34–41, 46] In this study, a widely known chemical mutagen ethyl methane sulfonate (EMS) [47, 48] was used to promote mutations in the strain. For preparing a selective medium for AA inhibition, glufosinate–ammonium (GA), L-methionine sulfoximine (MSO), and S-(2-aminoethyl)-L-cysteine (AEC) were applied in tests. This combination of bacteria, mutagen, and inhibitors was chosen from the results of a multicriteria analysis research performed by Berzina et al. [49].

## 2. Materials and Methods

### 2.1. Microorganism Strain

In this study, bacteria *B. subtilis* LMKK 897 was used. The microorganism was purchased from the Microbial Strain Collection of Latvia (MSCL). To prepare stock cultures, bacteria were cultivated in a 250-mL Erlenmeyer flask for 24 h in a nutrient broth liquid medium (peptone 5 g/L, meat extract 3 g/L, MnSO_4_ × H2O 0.01 g/L, pH 7) at 30°C, 150 rpm. Afterward, 0.5 mL of the culture was added to 0.5 mL of 50% glycerol solution in cryotubes. The tubes were stored in a freezer at −40°C for preservation.

### 2.2. Experimental Medium

For experiments, the following medium (molasses medium) was used: (NH_4_)_2_SO_4_ 2.5 g/L, sugar beet molasses (SIA “BIOferm”) 44.4 g/L, KH_2_PO_4_ 1.5 g/L, Na_2_HPO_4_ × 2 H_2_O 4.4 g/L, MgSO_4_ × 7 H_2_O 0.2 g/L, and trace element solution 1 mL/L and vitamin solution 1 mL/L. Trace element solution contained the following: MnSO_4_ × 4 H_2_O 0.192 g/L, CoCl_2_ × 6 H_2_O 0.237 g/L, H_3_BO_3_ 0.061 g/L, CuSO_4_ × 5 H_2_O 0.249 g/L, NiCl_2_ × 6 H_2_O 0.237 g/L, Na_2_MoO_4_ × 2 H_2_O 0.442 g/L, CaCl_2_ × 2 H_2_O 0.147 g/L, FeSO_4_ × 7 H_2_O 0.278 g/L, and KI 0.166 g/L. Vitamin solution contained biotin 0.01 g/100 mL and pyridoxine HCl 0.02 g/100 mL. The medium pH level was adjusted to 7.5, using 2 M NaOH and 5 M HCl. For agar plate preparation, 15 g/L agar was added to the medium composition.

It was important to use an inorganic nitrogen source ((NH_4_)_2_SO_4_) for this medium due to concern that if external proteins and AAs would be introduced through medium components, the inhibited strains would be able to uptake free AAs and circumvent the inhibitory effect of the AA inhibitors added to this medium in selection tests.

### 2.3. Determination of EMS Lethal Dose

The inoculum was prepared on the day before the experiment from the stock culture in the molasses medium described in Section 2.2. Method from three protocol examples was used with some adjustments [50–52]. Five EMS (Sigma-Aldrich, CAS 62-50-0, PN-M0880) concentrations were tested: 0 g/L, 10 g/L, 20 g/L, 40 g/L, and 60 g/L. Incubation with EMS was performed for 1 h at 30°C, 200 rpm in 50-mL tubes. After treating the culture with EMS, the culture was washed once with 5% Na_2_S_2_O_3_ and twice with 50 mM phosphate buffer (4.67 g/L K_2_HPO_4_, 3.15 g/L KH_2_PO_4_). Cells were centrifuged in between washes at 22°C 4000 rpm. The centrifuged cell pellet was then resuspended in the molasses medium and transferred to a 50-mL flask. The cell concentration was determined by cell counting using a hemacytometer, and dilutions were made so it was possible to inoculate 300 cells per plate. For determining the lethality rate, the agar medium was used described in Section 2.2. The plates were incubated for 48 h in triplicate to determine the EMS lethality rate. The experiment goal was to determine EMS concentration, which had a 50%–90% lethality rate.

### 2.4. Determination of AA Inhibitor Dose

For determining the inhibitor doses that ensure 100% inhibition, tests were performed using 48-well microplates (SARSTEDT, LOT 2021121) (working volume 0.5 mL), and OD600 measurements were made using a Tecan Spark multimode microplate reader. The following measurement parameters were set in SparkControl Magellan software to perform the OD600 measurements. The measurements were performed by inserting the microplate in a humidity cassette and placed in the microplate reader. Then, the microplate reader performed shaking at 216 rpm for 2 min and took the OD measurements. Then, the microplate was removed and placed in an incubator on an ELMI orbital shaker (DOS-20L) at 30°C, 300 rpm until the next measurement. The method described by Raita et al. for determining inhibition doses was modified for these experiments [52]. The inoculum concentration for each well was 1 × 10^6^ cells/mL. For each inhibitor, 16 doses were tested. Controls and each dose were in triplicate. For GA (Combi-Blocks, CAS 77182-82-2, batch: B30776), doses were in the range of 1 μM–0.4 M, for MSO(Sigma-Aldrich, PN M5379, CAS 15985-39-4)—0.01 mM–0.05 M, and for AEC (Sigma-Aldrich, PN A2636, CAS 2936-69-8)—0.025 mM–0.2 M. OD_600_ measurements were taken three times per day, and after 48 h, the cell concentration in samples was determined using a hemacytometer to supplement the microplate reader OD_600_ results and determine the inhibitor concentration that gives the complete inhibition of the growth of the wild-type (WT) strain. On the last day of the experiment, the cell concentration was determined for one sample from the triplet under the condition that its OD_600_ result did not deviate from the other triplet.

### 2.5. Obtaining and Comparing Mutant Candidates

After determining the EMS and inhibitor concentration for mutagenesis, these two factors were combined in one test where WT strain was treated with 40 g/L EMS and then selected on the molasses medium containing 0.05 M to 0.1 M GA or 0.025 M to 0.05 M MSO. The test was performed using 48-well microplates and a Tecan Spark multimode microplate reader using the same setup parameters as described in Section 2.4. There were multiple controls for the test: untreated WT in the medium without inhibitor (K1), treated WT in the medium without the inhibitor (K2), and untreated WT in the medium with inhibitors (K3). Each control was in triplicate. The rest of the wells were filled with medium supplemented with inhibitors and strain treated with EMS. Samples with a treated strain that had an OD_600_ value higher than the control with the same inhibitor (K3) were picked for further screening. Those cultures were transferred to a new 48-well microplate: the first two times in a ratio of 1:1 (culture: medium with inhibitor) and the last transfer culture to medium proportion in a ratio of 1:4. These potential mutant candidates were transferred to a new plate at least three times before they were stored and compared.

The candidates were compared with each other and with the WT strain in a shake flask test. They were cultivated in 50 mL of molasses medium in 250-mL microbial flasks at 30°C, 200 rpm, 48 h in triplicate. The molasses medium was used as described in Section 2.2.

To determine culture growth, OD_600_ was measured twice per day using the preprogrammed OD_600_ method on a spectrophotometer BioMate 160 (Thermo Scientific) with Genesis software. On the day of inoculation, the OD_600_ of the medium was measured, and the value was subtracted from further measurements. If the OD_600_ value was higher than 0.8, the samples were diluted and measured again. The results were calculated by multiplying dilution with the measured OD_600_ result. At the 48-h mark, the biomass samples were gathered in 50-mL tubes and centrifuged at 5000 rpm (3416 ×g) for 15 min. After the supernatant was removed, the tubes with biomass were inserted in a drying cabinet at 105°C for 24 h. The dry biomass was used for protein analysis.

From mutant candidate inoculants, strains were also stored at −40°C so that cultures are preserved for future tests.

### 2.6. Determination of Protein Concentration

To determine the protein concentration, a biomass of 0.01 g from each dry sample was weighted (±0.001 g). For cell lysis, a modified Guerlava, Izac, and Tholozan protocol [53] was performed. The samples were washed using 0.05 M MOPS ((3-[N-morpholino]) propane sulfonic acid) solution (1 mL/sample) and centrifuged at 16,000 ×g for 10 min 5°C. Afterward, 0.9 mL of supernatant was removed and 1 mL of NaOH–SDS solution was added to the sample for lysis (2.5 g SDS/100 mL and 0.8 g NaOH/100 mL). Next, the samples were inserted in boiling (100°C) water for five minutes. Afterward, they were cooled, vortexed till solubilized, and used to prepare a sample with a sample concentration of 0.75 mg/mL. This sample was used for protein analysis using bicinchoninic acid (BCA) protein kit (Pierce BCA Protein Assay Kit, PN: 23225) that was used according to the product assay protocol [54]. OD measurements were performed in a spectrophotometer using the BioMate 160 preprogrammed “Protein BCA” protocol.

### 2.7. AA Profile Identification Using High-Performance Liquid Chromatography (HPLC)

20 to 50 mg of the biomass was hydrolyzed in 6N hydrochloric acid with the addition of phenol at 110 ± 2°C for 24 h. Determinations of methionine (as a methionine sulfone) and the total content of cysteine and cystine (labeled in the text as Cys), measured as cysteic acid, were conducted after preoxidation of the samples with performic acid for 16 hours at 0°C prior toacid hydrolysis. AA analysis was performed using the protocol [55] with minor modifications. Tryptophane was not determined in this study.

Identification and quantification of AA contents were done using AA standard mix (A9781), methionine sulfone (> 99.0%), and cysteic acid (> 99.0%). Protein hydrolysates were derivatized automatically in-needle with o-phthalaldehyde (OPA) in the presence of mercaptopropionic acid (MPA), and 9-fluorenylmethyl chloroformate (FMOC) [56].

HPLC analysis was performed on the HPLC Shimadzu Nexera series using a reverse-phase YMC-Triart C18 column (150 mm ∗ 3.0 mm, 3 μm) with a guard column of the same type in the gradient elution mode. The chromatography conditions were as follows: mobile phases: A—20 mM potassium phosphate buffer pH 6.9; B—acetonitrile: methanol: H2O = 45:40:15 (v/v/v); flow—0.7 mL/min; column temperature—35°C; and inj. volume—0.3 μL. Gradient program was similar to described in Ref. [57]. The detection of derivatized AAs was done on two channels: Ex.-350 nm, Em.-450 nm; Ex.-266 nm, and Em.-350 nm (for proline). Accuracy determination of AA contents is approx. 10% relative.

To determine the protein quality AA score (AAS) and essential AA index (EAAI), the method described by Machado et al. [58] was used. The AAS evaluates the content of individual essential AA in the SCP relative to a reference protein [58, 59]. For reference, protein fish meal was used [60], which is considered the best source of high-quality protein with balanced AA content. However,the industry's unsustainability necessitates finding a substitute [61, 62]. It was calculated for 9 of the 10 essential AA for fish diet. The EAAI value characterizes the quality of the protein as follows: The protein is considered high quality when the value is 90% or higher, moderate quality when the value is from 70% to 89%, and low quality when the value is less than 70% [58, 63].

### 2.8. Strain Stability Test

Two of the gained protein-synthesizing mutants will be subcultured for 10 generations, thus testing genetic stability. The test was performed by cultivating the strains as in a shake flask test—cultivated in 50 mL of molasses medium in 250-mL microbial flasks at 30°C, 200 rpm in the molasses medium described in Section 2.2. After approximately 24 h, the cell density was determined, and measurements of biomass were taken. Afterward, a sample was transferred to a new flask so that the concentration in it is 1 ∗ 10^6^ cells/mL (same as inoculum). The rest of the flask content was centrifuged in 50-mL tubes and dried. The dry biomass was used for the protein analysis. However, the new flasks were inserted in the incubator. This was repeated for 10 days. For each generation, the biomass and protein concentration will be measured, and the cell density will be determined. This will help evaluate the strain genetic stability over successive generations.

### 2.9. Data Processing

**Figure 1:**
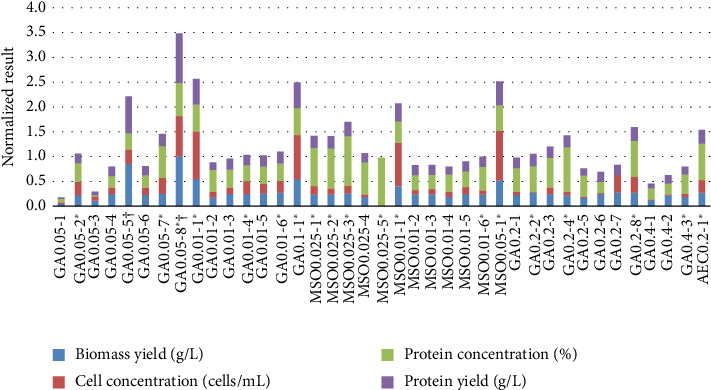
Comparison of mutant candidates to wild-type using scaled parameters of biomass yield, cell density, protein content, and protein yield. Scaling was performed using the min–max normalization method described in 2.8. The vertical axe depicts the summed scaled parameters of mutant subtraction to wild type. (⁣^∗^samples that were selected for HPLC analysis, and ^†^the biomass contained spores that did not settle down after centrifugation, therefore affecting and showing higher biomass yield than the other samples).

Data processing was performed using Microsoft Excel software (Microsoft Corporation). For EMS lethal dose, biomass yield, and protein content, the results were gathered in triplicate. Cell concentrations were determined for one sample from a triplet provided that the triplet contained no measurements that needed to be excluded from OD_600_ measurements. For Figure 1, parameters biomass production (g/L), cell density (cell number/mL), protein content (%), and protein production (g/L) were chosen to compare obtained mutants to WT *B. subtilis*. WT parameter values were subtracted from the mutant values, and the results were scaled using the min–max normalization method [64]. The biomass yield and protein content were statistically analyzed through a one-way ANOVA followed by Tukey's honestly significant difference. The same was done for the stability test results. Biomass and protein content data were also analyzed with principal component analysis (PCA) using Minitab software (Minitab, LLC (2021) (*Minitab*. Retrieved from https://www.minitab.com). For Pearson correlation, optical density (OD) data were used to analyze growth patterns using MATLAB software (MATLAB (2010), version 9.7.0.1 (R2019b), Natick, Massachusetts: The MathWorks Inc.).

## 3. Results and Discussion

### 3.1. EMS Lethality Rate

**Figure 2:**
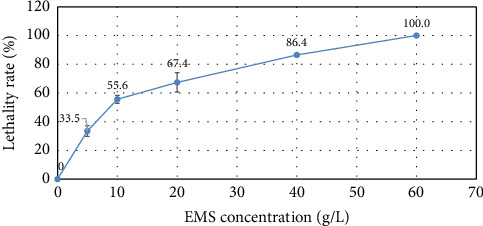
Lethality rate depending on the EMS concentration.

Mutating and screening microorganisms for industrial applications are crucial for developing enhanced strains successfully. EMS as a mutagenic produces random mutations in DNA by nucleotide substitution and typically induces point mutations [50]. To create a microorganism strain mutant, it is necessary to determine in what concentration the mutagen is lethal. For *B. subtilis,* five EMS concentrations were used to determine the lethality rate, and the results are displayed in Figure 2.

From the results (Figure 2), it is observable that *B. subtilis* exposure to EMS for 1 h at 10 g/L to 40 g/L concentrations provides the necessary lethality (50% – 90%). These results are comparable to what Ozdemir and Demirkan gained in their study when strain *B. subtilis* E6-5 was used. Their results showed 66% lethality at 20 g/L EMS, 80% at 40 g/L, and 90% at 60 g/L [50]. For other bacteria such as *Escherichia coli* and *Lactobacillus delbruecki*i, lethal EMS concentrations (> 99%) have been reported at 49.7 g/L [65] and 54.2 g/L [66], respectively. For *B. cereus* 31.04–62.08 g/L, EMS provided 94.80%–99.97% lethality [67]. Data indicate that the strain of *B. subtilis* used in the publication has similar EMS tolerance as other similar organisms. The results of the one-way ANOVA show a significant difference in lethality rates at different EMS concentrations, with an F-statistic of 231.45 and a *p* value of 1.69 × 10^−11^. Since the *p* value is much smaller than 0.05, we can conclude that there is a statistically significant difference in lethality rates between the EMS concentrations.

### 3.2. Inhibitor Dose

The inhibitor should promote complete growth inhibition on the WT strain so that it is most likely that a mutant with improved properties will be obtained on this selective medium. For these experiments, three different AA inhibitors were used—GA, MSO, and AEC. GA is a glutamine synthetase (GS) blocker that attaches to the glutamate binding site. GS is an enzyme involved in nitrogen metabolism and the production of glutamine, purines, and pyrimidines. Glutamine serves as a precursor in the biosynthesis pathways of the AAs: arginine (Arg), proline (Pro), aspartate (Asp), lysine (Lys), methionine (Met), threonine (Thr), and isoleucine (Ile). GA has been known to cause ammonia assimilation disorders and ammonia accumulation in prokaryotes [68]. This could lead to errors in mutant selection, as mutants with enhanced ammonia tolerance might be selected as false positives instead of those with improved glutamine and glutamine-derived AA synthesis capabilities [3]. MSO also inhibits GS; it is phosphorylated by GS, producing a transition state analog that remains bound to the active site, thus inhibiting the enzyme [69].

AEC inhibits two enzymes involved in AA synthesis—aspartate kinase (AK) and dihydrodipicolinate synthase (DHDPS) [70], which are involved in the biosynthesis of methionine (Met), lysine (Lys), threonine (Thr), and isoleucine (Ile) [3]. AEC is a toxic lysine analog and is known to inhibit bacterial growth [71–77].

**Figure 3:**
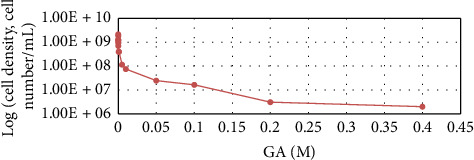
*B. subtilis* cell concentration depending on the GA dose after 48-h cultivation.

**Figure 4:**
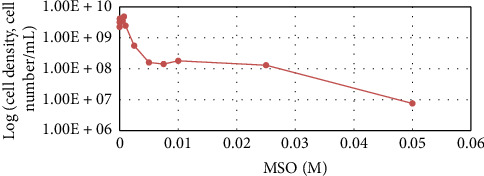
*B. subtilis* cell concentration depending on MSO dose after 48-h cultivation.

**Figure 5:**
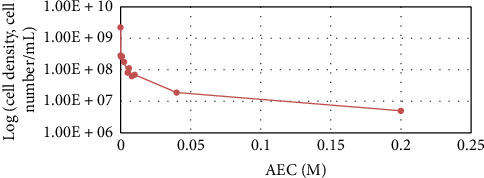
*B. subtilis* cell concentration depending on the AEC dose after 48-h cultivation.

The results of the AA inhibitor dosage effect on WT strain growth are displayed in Figures 3, 4, and 5.

From the results, it can be seen that partial growth inhibition was achieved when GA, MSO, and AEC inhibitors were used with doses of 0.1 M, 0.05 M, and 0.2 M, respectively. Compared to Shiio's research, which reported 100% growth inhibition using AEC for *B. subtilis* at a 6 mM dose [78], the strain used in this study compared to Shiio's has a much higher tolerance to the inhibitor. This is the first study that explores that GA and MSO inhibitors affect *Bacillus* spp. growth, but these inhibitors have been used with bacterial species such as *Mycobacterium tuberculosis* and *Azospirillum brasilense* [79, 80]. GA provided 50% inhibition for *M. tuberculosis* with 1.5 μM [80] and MSO provided 50% inhibition with 0.05 mM [79]. MSO for *A. brasilense* provided 100% inhibition with a 0.028 mM dose [79]. *B. subtilis* as seen in figures does not have growth inhibition in these concentrations. For this bacterium, much higher concentrations are needed showing its high tolerance and endurance in stress-inducing conditions.

As mentioned before, the inhibitors in this study were chosen according to a multicriteria analysis study by Berzina et al. [49]. Even though these three inhibitors had the closest to ideal result, other AA inhibitor effects also have been tested for *Bacillus* spp. The inhibitor glyphosate has been the only one reported that has shown 100% growth inhibition at a 1.8 mM dose [81], even though some of the *B. subtilis* strains are capable of glyphosate degradation [82]. Other inhibitors such as metsulfuron-methyl at 0.03 mM dose caused 50% inhibition [83], and imazethapyr and imazapyr at 0.1 mM dose did not induce inhibition but slowed growth speed [72, 84]. According to Florlani et al., chlorsulfuron at 0.03 mM dose can cause a significant inhibition for *B. subtilis* [72].

*B. subtilis* is known for its high tolerance to various stresses and ability to adapt to harsh environments [85, 86]; therefore, it should not be surprising that high doses of inhibitors are needed*. B. subtilis* is capable of herbicide degradation; therefore, their use for successful AA inhibition might be challenging [82, 87]. *B. thuringiensis* can produce proteins that are toxic to insects, which is used for the production of microbial pesticides [88].

### 3.3. Obtaining Mutant Candidates

**Figure 6:**
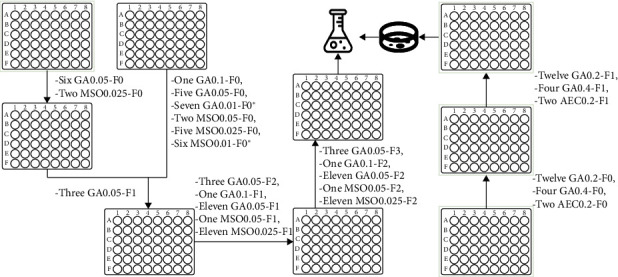
Scheme of obtaining *Bacillus subtilis* mutants (∗—mutant candidates were transferred to a well with higher inhibitor concentration, F0–F3—number of transfer).

After the WT strain was treated with EMS, it was inoculated in the molasses medium and separated into microplate wells with inhibitors. The inhibitors created selective pressure, which theoretically should only allow the development of mutants that have acquired the desired property of synthesizing more AAs.  Figure 6 shows the progress of how many potential candidates were selected for each transfer. The candidates were selected by comparing the growth rate to the control, as increased protein and AA concentrations should allow the candidate to withstand the inhibition and grow in the selective medium. In Figure 6, GA0.1 has dose 0.1 M, GA0.05—0.05 M, GA0.01—0.01 M, MSO0.01—0.01 M, MSO0.025—0.025 M, MSO0.05—0.05 M, GA0.2—0.2 M, GA0.4—0.4 M, and AEC0.2—0.2 M. After cultivating the samples in the last microplate, together 27 samples were transferred to 50-mL flasks in molasses medium and cultivated overnight. Eighteen candidates selected using inhibitors GA (dose 0.2 M and 0.4 M) and AEC (dose 0.2 M) were transferred on Petri plates. Later, these candidates were used to prepare inoculants for a shake flask test. These were used as inoculums in shake flasks test and for short-term storing of candidate strains.

### 3.4. Comparison of Mutant Candidates

The potential mutant candidates were analyzed and compared in a shake flasks test in which various parameters were obtained: biomass yield, cell density, protein content, and yield, and for those to whom AA content was determined, the AAS was also calculated. The results are shown in Figure 1 and Table 1.

The majority of obtained mutants showed improvements in biotechnologically important parameters such as biomass yield, cell density, protein content, and yield. It can be seen that GA0.05-5, GA0.05-8, GA0.01-1, GA0.1-1, and MSO0.05-1 were significantly superior compared to the WT with the largest overall difference. Although GA0.05-5 and GA0.05-8 had measurement errors for biomass yield, these samples still presented higher cell concentration and protein concentration than other samples. Overall, the mutants showed great improvements in biomass production, cell density, and protein production. Multiple mutant candidates stood out with one or multiple parameters that were higher than the WT strain. Biomass yields were lower than the WT only for two mutant candidates GA0.05-1 and MSO0.025-5. The cell concentration and protein content for all mutant candidates were higher than the WT strain. The protein yield for all candidates was higher than the WT with one expectation-MSO0.025-5, which, despite the high protein concentration, due to the low biomass yield is also low in protein yield. These results indicate that culturing the strain in selective media allowed to select mutants with better fermentation results than their WT strain. The sample results can be viewed in Table 1.

Kurbanoglu and Algur in their study obtained a growth rate of *B. subtilis* in batch fermentation of 0.15 g/L/h and a protein production rate of 0.11 g/L/h [10], which is five times higher than the WT strain in this study. *B. subtilis* should be able to reach up to 71% of protein content in the biomass [10], but in this experiment, none achieved this result. It should be noted that the mentioned researcher used ram horn hydrolysate as a substrate to grow the microorganism. That substrate already contains AAs, and when comparing inorganic with organic nitrogen sources, organic sources are easier for microorganisms to assimilate [89] and synthesize proteins. Nitrogen source can be one of the most important factors that can directly influence protein synthesis by microorganisms [90]. In this experiment, the strains only had (NH_4_)_2_SO_4_ as a nitrogen source. Inorganic nitrogen sources are relatively abundant and available at low cost in most countries; consequently, it is cheaper to use them in fermentation. In these first experiments, a medium without organic nitrogen was used, because the inhibitory effect is better observed in media containing an inorganic nitrogen source absent from proteins and AA causing microorganisms in the selection stage to compensate for the lack of them.

After these results, (NH_4_)_2_SO_4_ was replaced with peptone (5 g/L) and meat extract (3 g/L) as nitrogen source influence on biomass and protein yield needed to be analyzed. Knowing that each microorganism responds in its own way to different nitrogen sources and concentrations this factor should be optimized. In future research, medium with (NH_4_)_2_SO_4_ should be optimized by finding adequate carbon and nitrogen ratio and partly replacing the inorganic nitrogen with a waste substrate, for example, liquid cheese whey. It should be noted that if the mutant strain has been grown on media that already contains organic nitrogen, then the strain should not be stored or reused from this media. There is a possibility that the improved protein-producing properties of the strain will be lost. Mutant microorganisms have better chances of keeping their gained qualities if used under environmental conditions corresponding to the employed selective stressor [91]. Otherwise, those abilities can decline in time and may eventually be lost [88, 91]. When selecting mutants, it is important that there is no organic nitrogen in the culture medium to select those microorganisms that are capable of synthesizing AA themselves. If the AAs are in the medium, then the organism is no longer under pressure to synthesize them by itself and will use the available ones. They can adapt to new conditions in which they do not need to work as much and lose the acquired mutations [88].

The highest biomass results were gained by GA0.01-1, GA0.1-1, and MSO0.05-1 mutant candidates, which led to the highest biomass conversation ratio (up to 0.2 g/g_subst_) and protein yield (up to 4 g/L). GA0.01-1 and GA0.1-1 on average are 7.1 times higher biomass yield, 21.2 times higher cell concentration, 1.4 times higher protein content, and 10.2 times higher protein yield than the WT. The samples GA0.05-5 and GA0.05-8 as noted under Table 1 contained spore sediments that did not settle down after centrifugation, making it difficult to decant them. Therefore, these samples contained more residual medium than the others and were hard to compare. The cell concentration and protein content would be the most objective parameters when comparing these two samples to the others. Both these samples gained a higher cell concentration than most candidates (8.33 ∗ 10^9^ and 2.04 ∗ 10^10^), and GA0.05-8 also achieved a high protein concentration (51.03 ± 2.7%).

GA0.05-8, GA0.01-1, GA0.1-1, GA0.2-7, GA0.2-8, MSO0.01-1, and MSO0.05-1 may have gained a mutant variation that allows them to grow in higher cell density than the other candidates. This provides them with the ability to gain higher biomass and protein yields, significantly improving the amount that can be obtained in single fermentation. In general, each group of inhibitors has at least one candidate mutant that stands out for some property. These candidates, GA0.05-8, GA0.01-1, GA0.1-1, GA0.2-4, MSO0.01-1, and MSO0.05-1, each in their groups stood out with higher protein concentrations ranging from 1.4 to 1.6 times higher than WT. The MSO0.025 group has several candidates that show the improvement of this parameter and altogether this group stood out with increased protein concentration. In other groups, it is evident from the comparison of candidates that the percentages of protein content are roughly similar with some exceptions. GA0.05-7, GA0.05-8, GA0.2-4, and GA0.2-8 showed higher protein contents than the other GA candidates in their respective groups. The only mutant candidate that showed poorer results than the WT for biomass yield, cell concentration, and protein yield was MSO0.025-5. However, this candidate stood out above others with the high darker biomass color and its high protein content (56.70 ± 3.8%). The results indicate that already at this point of the study, it can be seen that with the proposed method, it is possible to improve the amount of biomass and protein concentration in the strain.

Mutants Ga13-3, GA0.2-7, and GA0.05-2 showed the least statistically significant correlations with other mutants and also lacked a significant correlation with the WT strain. This observation suggests that these mutants may have acquired unique traits through mutagenesis.

**Figure 7:**
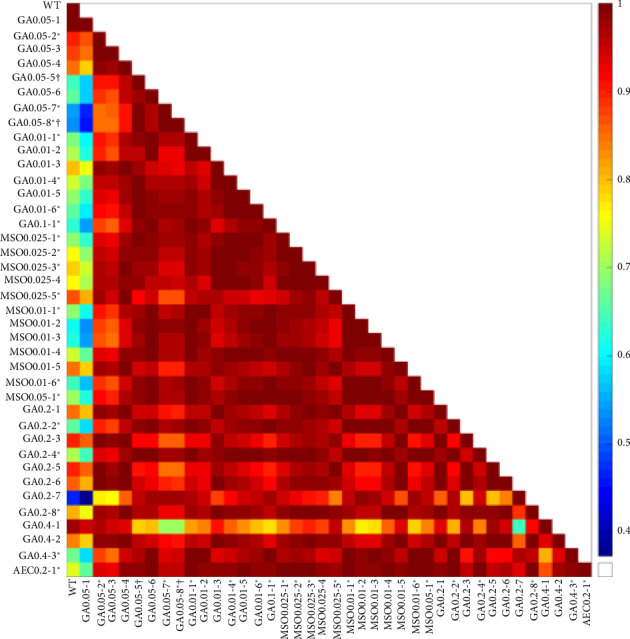
Pearson coefficients of correlation between mutants and wild-type *B. subtilis*. Pearson coefficients were acquired using pairwise analysis.

**Figure 8:**
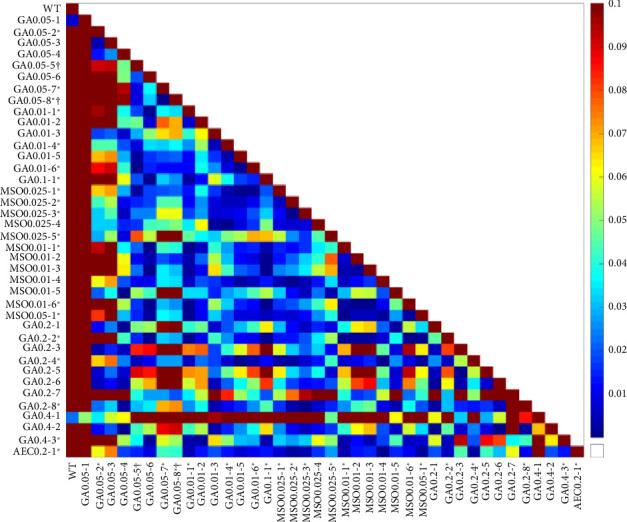
*p* values of correlation between mutants and wild-type *B. subtilis*. *p* values were acquired using pairwise analysis.

To compare the acquired changes in growth patterns, the OD data of mutants and WT *B. subtilis* were analyzed using Pearson correlation (as shown in Figures 7 and 8). Almost all mutants exhibited high Pearson correlation coefficients with each other, falling within the range of 0.85–1.0. These correlations were statistically significant, with a *p* value of around 0.02 (Figure 8).

However, mutants GA04-1 and GA0.05-1 had small Pearson correlation coefficients with other mutants but significant coefficients with the WT *B. subtilis*, suggesting that these mutants were more similar to the WT than to other mutants. The remaining mutants did not exhibit statistically significant correlations with the WT, indicating their dissimilarity (Figure 8).

Mutants Ga13-3, GA0.2-7, and GA0.05-2 showed the least statistically significant correlations with other mutants and also lacked a significant correlation with the WT strain. This observation suggests that these mutants may have acquired unique traits through mutagenesis.

**Figure 9:**
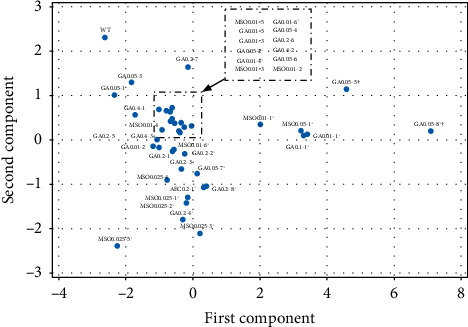
Principal component analysis (PCA) of mutants and wild-type *B. subtilis*.

Biotechnologically important parameters, including biomass yield, cell density, protein content, and yield of mutant and WT *B. subtilis,* were analyzed with PCA (see Figure 9). The analysis did not reveal distinctive clusters, suggesting that the inhibitor type does not exhibit selectivity for these parameters. Similar to the Pearson analysis, PCA indicated that mutants differ significantly from WT *B. subtilis*, as no cluster formed around the latter. Notably, PCA identified a few unique mutants, such as GA0.05-8, GA0.05-5, and MSO0.025-5.

WT, GA0.05-2, GA0.05-7, GA0.05-8, GA0.01-1, GA0.01-4, GA0.01-6, GA0.1-1, MSO0.025-1, MSO0.025-2, MSO0.025-3, MSO0.025-5, MSO0.01-1, MSO0.01-6, MSO0.05-1, GA0.2-2, GA0.2-4, GA0.2-8, GA0.4-3, and AEC0.02-1 were selected for AA determination via HPLC. The AAs analyzed were aspartic acid (Asp ac), glutamate (Glu ac), serine (Ser), glycine (Gly), alanine (Ala), tyrosine (Tyr), cysteine (Cys), proline (Pro), arginine (Arg), histidine (His), isoleucine (Ile), leucine (Leu), lysine (Lys), methionine (Met), phenylalanine (Phe), threonine (Thr), and valine (Val). The HPLC results for *B. subtilis* WT and mutant candidate AA profile are represented in Tables 2 and 3.

Every mutant candidate had obtained higher protein content than the WT. MSO and GA are the inhibitors of glutamine synthetase, an enzyme involved in nitrogen metabolism and the synthesis of glutamine, purines, and pyrimidines. It acts as the metabolic signal for nitrogen availability [92]. Knowing that glutamine is a precursor to the biosynthesis pathway of the AAs Arg, Pro, and Asp, as well as AAs of aspartate origin—Lys, Met, Thr, and Ile, it was more or less expected that these AA profiles would be changed [80, 93, 94]. AEC is an aspartate-derived AA inhibitor and theoretically inhibits the biosynthesis of Met, Lys, Thr, and Ile [3]. The HPLC results for candidate mutants are represented in Tables 2 and 3.

The mutant candidates selected with GA inhibitor showed an increase in Asp ac, Ser, His, Gly, Thr, Arg, Val, Met, Phe, and Leu. For Ala, Tyr, Ile, Lys, and Pro only in one or two candidates, a decrease is visible. According to the HLPC results, the WT protein content was 43.7%, and for the mutant candidates, it was 48.2% and above except for the GA0.05-8 candidate, for which this value was 44.4%. All of the mutants selected with GA inhibitor have a higher protein content than the WT strain and the EAA/NEAA (essential AA/nonessential AA) ratios are higher than the WT strains except for GA0.05-8, which had a lower result by 0.01 g/100g_sample_. On average, the GA inhibitor increased the protein concentration by 7% and in three out of 11 cases mutant candidate has gained an EAA/NEAA higher than 0.9. These results already indicate that with the used method, strains with improved protein-synthesizing properties and AA profile have been selected.

Seven out of 11 mutant candidates that were selected with GA inhibitor have gained at least 20% higher Met content g/100g_sample_. It is the first-limiting AA in plants and rendered animal proteins, and it is involved in various metabolic pathways and many physiological processes [95]. Met plays an important role in the synthesis of other metabolites, can improve feed utilization, and is considered indispensable [95–97]. Due to its significance and limited presence in traditional protein sources, mutants with increased Met content have a higher potential of being used as valuable feed [3].

In Table 2, there are results for WT and WT2. WT2 is the same sample that was reanalyzed. This was done to perform repeated sample hydrolysis and injection into the chromatograph to determine how sensitive the analysis is. From the data, it can be seen that the results in these separate analyses are quite similar. The difference ranges from 0.5% (Phe) to 25.7% (Met). The average difference for the AA results is 4.6%, indicating that they have good repeatability. Similarly, WT, GA0.1-1, and MSO0.01-1 strains were cultivated in independent tests to compare whether the AA profile results have repeatability. AA results differed on average by 10.6% for WT, 4.8% for GA0.1%-1%, and 13.1% for MSO0.01-1, indicating that there may be differences between individual tests, but these are within acceptable limits. Most importantly, in these separate tests, the results showed that the selected mutants have higher AA yields than the WT, confirming that the method used works for mutant selection.

Similarly, as for candidates selected with GA inhibitor, those that were obtained by using MSO inhibitors show an AA increase for Asp ac, His, Gly, Thr, Arg, Val, Met, Phe, Ile, and Leu. For AA as Glu ac, Ser, Ala, Tyr, Lys, and Pro, only one or two mutant candidates showed a decrease. For the one mutant gained using AEC inhibitor, the only AA that has not increased is Cys. All of these candidates show a higher protein content than the WT strain. Only one MSO0.025-2 showed an EAA/NEAA ratio lower than the WT. In two out of seven cases, the EAA/NEAA ratio is higher than 0.9. In general, mutants have either improved EAA/NEAA ratio or protein concentration, or both, confirming the assumption that improved mutant strains can be obtained with the proposed method. On average, the MSO inhibitor increased the protein concentration by 9%. From the AA profiles of the mutant candidates, it can be observed that the amount of AA has increased, both in those that should have been theoretically affected and in others. AA synthesis is a complex process, and by putting pressure on the synthesis of some AA, it can be expected that the synthesis of others can be affected too. This is also expected because of the increase in the crude protein concentration in the samples. It should be noted that the determination of Cys is difficult under reverse-phase chromatography conditions. The peak of highly polar cysteic acid overlaps with the peak of aspartic acid after preoxidation, and it is difficult to resolve those two compounds. Nevertheless, the amount of Cys was evaluated as a difference between peaks of aspartic acid from oxidated and nonoxidated samples (also taking into account differences in sample weights token for analysis). That is why the Cys content was not determined with accuracy. For this reason, it is difficult to estimate the effect of inhibitors on Cys synthesis and to conclude whether they contributed to the increase or decrease of this AA. Similar difficulties occur for the determination of Met—the peak of Methionine sulfone is partially overlapped with Arg, which decreases the accuracy.

Unfortunately, with AEC inhibitor in these experiments, only one mutant candidate was selected. Although it has an improved AA profile, more mutants should be gained to objectively evaluate the effect of the inhibitor.

To assess the quality of *B. subtilis* mutant strains as a protein source for animal feed, fish feed was used as a reference protein in the calculations of AAS and EAAI [60]. The concept of assessing protein quality based on a protein's constituent AA is recommended by expert committees of the World Health Organization (WHO), United Nations Food and Agriculture Organization (FAO), and others, for routine evaluation of protein quality [98]. The AAS shows how efficiently protein will meet a person's or animal's AA needs by comparing the concentration of the first-limiting essential AA in the test protein with the concentration of that AA in a reference pattern [99]. The first-limiting AA has an important role in determining the relative value of the dietary protein and is used as a reference when substituting protein sources with an equivalent amount [63]. The AAS is calculated for each AA. The results are represented in Table 4 (% < 100%—lower than reference, % < 100%—higher than reference).

When comparing WT and *B. subtilis* mutants by essential AAS and by their first-limiting AA, none exceed the quality of fishmeal. For most of the mutants, the first-limiting AA is Met or Lys. GA0.05-8 was classified as a low-quality protein, while GA0.2-8 was classified as a high-quality protein, and the other candidates were classified as moderate-quality proteins according to AAS. Comparing the AAS for individual AA with referenced fish meal for all samples indicates that Phe, Ile, and Leu contents are higher, and Lys and Met are lower, although some are close to the required quality. For example, GA0.2-8 mutant has gained AAS 91.5%, which classifies it as a high-quality protein. Although gained mutants have better AA profiles than the WT strain AAS comparison of GA0.05-8, GA0.2-2, MSO0.01-6, and MSO0.025-5 indicates that they are of lower quality. It should be remembered that this is only according to their limiting AAs, namely, according to the AA that has the lowest amount in the sample, and the obtained mutants with other AAs exceeded the amount in WT.

Comparing overall profiles for those mutants that were selected using the GA inhibitor and MSO inhibitor shows that by using the GA inhibitor gained mutants acquire more significantly improved strain qualities than those selected with MSO. If there were more AEC inhibitor-selected mutants, then a similar conclusion would need to be developed.

**Figure 10:**
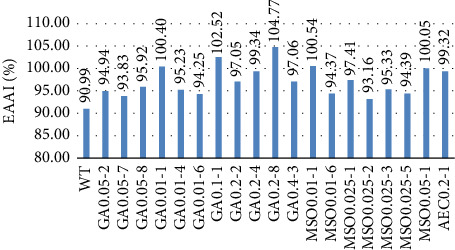
Essential amino acid index of *B. subtilis* and its mutant candidate.

EAAI is the overall protein quality of the food, which is the geometrical mean of the ratio of all EAA in the evaluated protein relative to their content in a highly nutritive reference protein such as fishmeal [63, 100]. This parameter reflects more on the biological quality of a protein than AAS [58]. The results of EAAI are represented in Figure 10 (% < 100%—lower than reference, % < 100%—higher than reference).

Using EAAI as a quality indicator, all mutants classify as high-quality proteins and GA0.01-1, GA0.1-1, GA0.2-8, MSO0.01-1, and MSO0.05-1 can be considered similar to the reference protein fish meal from their overall protein quality. Referring to the flask test results, mutants also gained high biomass yields and cell concentrations except for the GA0.2-8 candidate, which had moderate biomass but high cell concentrations and protein content. GA0.2-8 mutant had 3.7 times higher biomass content, 1.5 times higher protein concentration, 1.2 times higher AA content, and 1.2 times higher EAAI in comparison with WT. This candidate not only showed one of the highest Met values but also had the highest Lys content, which often is the limiting AA in proteins.

### 3.5. Strain Stability

**Figure 11:**
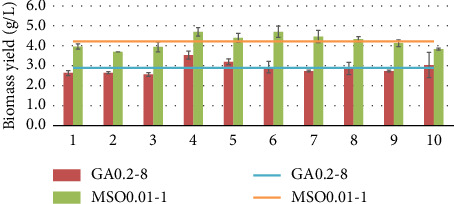
Genetic stability of GA0.2-1 and MSO0.01-1 mutants in terms of biomass yield.

**Figure 12:**
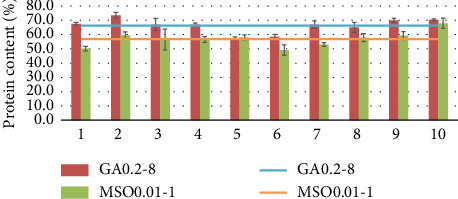
Genetic stability of GA0.2-1 and MSO0.01-1 mutants in terms of the protein concentration.

**Figure 13:**
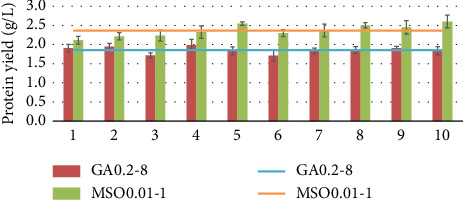
Genetic stability of GA0.2-1 and MSO0.01-1 mutants in terms of protein yield.

Two mutants GA0.02-8 and MSO0.01-1 were subjected to subculturing in a shake flask test for testing their genetic stability over 10 generations. These two strains were chosen because they had relatively high results compared to the others and to test both a single mutant obtained using GA and MSO as inhibitors. The results are presented in Figures 11, 12, and 13, where the results are shown for each generation (bars) and the average value of the results of these generations (line).

As shown in Figures 11, 12, and 13 after 10 passages, the strains maintained relatively consistent biomass and protein yields, indicating stable genetic traits. The average protein concentration for GA0.2-8 was 66.3%, and 56.8% for MSO0.01-1. One-way ANOVA followed by Tukey's honestly significant difference showed that there are no significant differences (*p* > 0.05) between each generation for mutants. The lack of significant differences across generations helps indicate that the traits remain stable and consistent in the mutant strains over time.

### 3.6. Discussion

Altogether, it should be concluded that it has been a success in obtaining mutants superior to WT, in terms of quality and capability of producing higher amounts of proteins and AA. GA, AEC, and MSO inhibitors can be successfully used to apply selective pressure and select mutants with improved protein and AA profiles. But in the future investigations with a larger total number of screened mutants should be conducted, as method limits remain unknown. The acquired mutants should be further improved and tested. It should be mentioned that a relatively small number of mutants were generated in this study, as the goal was to test whether the method works, not to generate the best mutant. Nevertheless, this study still demonstrates the method capability of improving strains. It would be necessary to determine the digestibility of the best mutants, how they would affect fish and other potential users when used in the diet, and the limits of how much can be used. To supplement the results, future research should analyze the mutants obtained through AA inhibitor-guided selection based on changes in their gene expression levels. Since many genes are responsible for protein synthesis, AA biosynthesis, and the corresponding metabolic pathways, the interpretation of RNA sequencing results could be extensive. Additionally, the expression levels of random mutants might significantly differ among mutants that practically showed very similar biomass, protein concentration, and AA index values in this study.

In the future, the best mutant candidate should be tested on a bigger scale, for example, in a 5-L bioreactor. It would be interesting to also compare the process parameters in batch, fed-batch fermentation, and continuous fermentation knowing that the WT *B. subtilis* has not been used in continuous fermentation for SCP production thereby research in this section could be beneficial. In theory, in a fed-batch or continuous fermentation, the results should be higher than in batch fermentation; this is indicated by previous studies, where *B. subtilis* has been used to produce other products [101–104]. Foam generation during a large-scale production could bring issues. An antifoam should be applied for this process [105–108], although it is often an unattractive solution since the challenge remains when upscaling to the industrial level [109]. Foam fractionation has been applied in surfactin production [110–112]. From a practical perspective, this medium is easy to prepare and would be practical in an industrial process. When using agricultural or industrial residues as substrates for SCP production, it can be difficult to implement them in the medium. Various substrates would need to be pretreated, which could be a crucial step in the technology [49], but molasses is easily incorporated into the medium. This substrate does not need to be pretreated, it is rich in sugars, and around 50% of it is sucrose and glucose, providing the microorganism with nutrients [113, 114]. In an industrial process, the medium would be easily prepared, by mixing and sterilizing it. Substrate hydrolysis or sediment separation would not be required. However, comparing obtained mutants to already established industrial microorganisms, it seems that *B. subtilis* mutants still need to be improved to become a feasible SCP producer. It could be whether optimizing the medium or optimizing the fermentation process. Abou-Taleb showed that fed-batch fermentation can increase the *B. subtilis* biomass results 1.6-fold compared to batch fermentation [12]. One of the leading microorganisms in SCP production is *Fusarium venenatum* as microorganism biomass is successfully commercialized for human consumption [115]. The biomass production for this microorganism in an air-lift system can reach 193.5 g/L/h [116], while the highest production rate of obtained mutants reaches only 0.2 g/L/h. It is noteworthy that *F. venenatum* is a filamentous fungus thus metabolism and physiology including production capacity are not comparable. Yeasts, for example, *Candida utilis* in batch fermentation can reach a biomass production rate of 0.68 g/L/h and in continuous fermentation biomass production rate can reach 1.62 g/L/h and a protein production of 0.63 g/L/h [117]. Therefore, applying a different cultivation type could be beneficial for increasing *Bacillus* kinetic performance in fermentation. High yield and good quality are necessary for SCP technology to be competitive. The improved strain could be also used in mixed culture fermentation. There have already been examples where *Bacillus* strains have been used together with other microorganisms, which would justify this possibility. In the study by Sheng et al., the cofermentation of citrus pomace with *B. amyloliquefaciens* and *C. utilis* resulted in significant improvements in crude protein, free AA, small peptides, and lignocellulose levels [19]. Similarly, in the study by Weng and Chen, soybean fermentation with *Rhizopus oligosporus* and *B. subtilis* resulted in an increased soluble nitrogen content, degree of hydrolysis of soy protein, and AA contents compared to single starter culture fermentation by *B. subtilis* alone [21]. These studies highlight the versatility and efficacy of two-step fermentation approaches in improving the nutritional content and SCP production.

It is important to mention that for mutants, the number of generations should be reduced as much as possible, so it has a better chance of keeping their acquired qualities [52]. Gained qualities can decline in time and may eventually be lost after multiple generations if they no longer grown under stressful conditions that create the relevant selective pressure [88, 91]. It has been previously observed that strains can lose the ability to produce products upon repeated transfers and the production rate can decrease during the long-time fermentation [88]. It is needed to avoid this possibility by storing the culture samples in cold storage and restoring them from time to time.

## 4. Conclusions

In this study, a novel approach to creating mutant strains with enhanced protein and AA content was experimentally validated. Previously, this approach has been only proposed theoretically by Spalvins et al. in the scientific literature [3]. This research shows that it is possible to create bacteria mutant strains by applying EMS chemical mutagen and AA inhibitors. Together 26 mutants were gained by applying GA as an inhibitor, 12 mutants with MSO, and one with AEC. The results demonstrate that GA, AEC, and MSO inhibitors can be successfully used to apply selective pressure and select mutants with improved protein and AA profiles. Gained mutants showed an increase in biomass yields, cell concentration, protein content, and protein yield. All except one mutant showed higher results than the WT, but this exception stood out for its high protein concentration (MSO0.025-5). The highest results gained in the shake flask test were by GA0.01-1 and GA0.1-1 (8.9 ± 0.3 g/L, 2.4 ∗ 10^10^, 48.7 ± 2.6%, 4.4 ± 0.34 g/L, and 8.9 ± 0.3 g/L, 2.2 ∗ 10^10^, 48.7 ± 2.6%, 4.4 ± 0.34 g/L, respectively). The results of these mutants on average are 7.1 times higher biomass yield, 21.2 times higher cell concentration, 1.4 times higher protein content, and 10.2 times higher protein yield than the WT. PCA indicated that there is no correlation between these parameters and inhibitor type but confirmed that mutants have clear statistical differences from WT *B. subtilis*. Also, the Pearson correlation showed mutant statistical differences from WT in growth pattern, indicating mutant dissimilarities from WT. For 19 candidates, AA profiles were also determined by HPLC. These results showed that the selected mutants compared to the WT strain have improved AA profiles and higher essential AA to nonessential AA ratio. Most of the candidates obtained methionine or lysine as the first-limiting AA. By determining the protein quality according to amino acid score (AAS), GA0.05-8 classifies as a low-quality protein, GA0.2-8—as a high-quality protein, and the other candidates—as moderate-quality proteins using fish meal as reference protein. However, by using the EAAI as a quality indicator, all mutants were classified as high-quality proteins and GA0.01-1, GA0.1-1, GA0.2-8, MSO0.01-1, and MSO0.05-1, reaching or even exceeding reference protein overall protein quality. The lowest EAAI was for the WT strain. This again shows that improved strains can be successfully selected by this method. The best-performing mutant GA0.2-8 was selected when using GA—7.1 times higher biomass content, 1.5 times higher protein concentration, 1.2 times higher AA content, and 1.2 times higher EAAI was achieved in comparison with WT.

Data from this study show that the proposed approach can generate novel mutants with higher AA content and protein amount. Thanks to this research, it is confirmed that we can create improved mutants with this method. As a result, future investigations with a larger total number of screened mutants should be conducted, as method limits remain unknown. The increased number of tested mutants should help us acquire even better mutants as mutants obtained from this study were not industrially competitive with existing solutions. Regardless, the purpose of testing the proposed novel technique was effectively met. In addition, more mutants should be selected using the AEC inhibitor to better determine its effect on selective pressure. Because this research's main goal was to validate the method, in the future, it could be possible to research the possibilities of combining inhibitors or modifying this principle and gain even better and more competitive mutant strains. The mutants should also be tested on bioreactors and in different cultivations. It would be important to note that there should be employed multiple preventive actions to sustain the enhanced abilities of the mutants as long as possible, so it does not lose the newly acquired qualities.

## Figures and Tables

**Table 1:** Biomass and protein yield results for *B. subtilis* and its mutant candidates from shake flask tests.

Code	Biomass yield (g/L)	Cell density (cell number/mL)	Protein (%)	Protein yield (g/L)
WT	1.3 ± 0.2	1.08 ∗ 10^9^	33.70 ± 1.1	0.32 ± 0.03
GA0.05-1	1.2 ± 0.3	2.38 ∗ 10^9^	40.19 ± 1.7	0.35 ± 0.07
GA0.05-2^a^	3.9 ± 0.3^c^	7.93 ∗ 10^9^	45.73 ± 0.9^d^	1.32 ± 0.14^e^
GA0.05-3	2.1 ± 0.1	3.75 ∗ 10^9^	39.18 ± 0.5	0.62 ± 0.03
GA0.05-4	4.2 ± 0.2^c^	4.90 ∗ 10^9^	42.87 ± 0.3	1.34 ± 0.07^e^
GA0.05-5^b^	13.7 ± 2.2^c^^b^	8.33 ∗ 10^9^	44.78 ± 1.9^d^	0.32† ± 0.97
GA0.05-6	3.9 ± 0.2^c^	5.18 ∗ 10^9^	43.29 ± 1.7^d^	0.35 ± 0.04
GA0.05-7^a^	4.3 ± 0.2^c^	9.18 ∗ 10^9^	50.56 ± 2.1^d^	1.32 ± 0.10^e^
GA0.05-8^ab^	16.0 ± 2.3^c^^b^	2.04 ∗ 10^10^	51.03 ± 2.7^d^	0.62† ± 0.44^e^
GA0.01-1^a^	8.9 ± 0.3^c^	2.37 ∗ 10^10^	48.71 ± 2.6^d^	1.34 ± 0.34^e^
GA0.01-2	3.1 ± 0.5	4.40 ∗ 10^9^	46.92 ± 3.1^d^	4.58 ± 0.18^e^
GA0.01-3	4.3 ± 0.2^c^	4.50 ∗ 10^9^	45.55 ± 1.8^d^	1.27 ± 0.14^e^
GA0.01-4^a^	4.2 ± 0.3^c^	7.75 ∗ 10^9^	44.66 ± 1.3^d^	1.61 ± 0.09^e^
GA0.01-5	4.5 ± 0.1^c^	6.03 ∗ 10^9^	45.26 ± 2.7^d^	6.08 ± 0.11^e^
GA0.01-6^a^	4.7 ± 0.2^c^	7.03 ∗ 10^9^	45.26 ± 1.1^d^	3.25 ± 0.04^e^
GA0.1-1^a^	8.9 ± 0.6^c^	2.20 ∗ 10^10^	48.77 ± 4.4^d^	1.09 ± 0.40^e^
MSO0.025-1^a^	4.1 ± 0.2^c^	5.63 ∗ 10^9^	52.94 ± 2.6^d^	1.46 ± 0.16^e^
MSO0.025-2^a^	4.1 ± 0.2^c^	4.45 ∗ 10^9^	53.53 ± 1.7^d^	1.40 ± 0.06^e^
MSO0.025-3^a^	4.4 ± 0.3^c^	5.38 ∗ 10^9^	57.11 ± 3.8^d^	1.51 ± 0.07^e^
MSO0.025-4	3.4 ± 0.8	2.83 ∗ 10^9^	50.61 ± 0.7^d^	1.60 ± 0.28^e^
MSO0.025-5^a^	0.4 ± 0.1	1.70 ∗ 10^9^	56.70 ± 3.8^d^	3.25 ± 0.05^e^
MSO0.01-1^a^	6.7 ± 0.5^c^	2.18 ∗ 10^10^	46.63 ± 1.7^d^	1.61 ± 0.25^e^
MSO0.01-2	4.1 ± 0.6^c^	3.73 ∗ 10^9^	44.30 ± 0.9^d^	1.65 ± 0.18^e^
MSO0.01-3	4.2 ± 0.3^c^	4.13 ∗ 10^9^	44.06 ± 1.2^d^	1.86 ± 0.12^e^
MSO0.01-4	3.4 ± 1.0	4.15 ∗ 10^9^	45.08 ± 1.5^d^	1.28 ± 0.32^e^
MSO0.01-5	4.2 ± 0.8^c^	4.92 ∗ 10^9^	44.60 ± 1.1^d^	0.25 ± 0.30^e^
MSO0.01-6^a^	4.0 ± 0.2^c^	3.70 ∗ 10^9^	47.46 ± 2.0^d^	2.33 ± 0.15^e^
MSO0.05-1^a^	8.5 ± 0.6^c^	2.46 ∗ 10^10^	48.17 ± 1.7^d^	1.37 ± 0.18^e^
GA0.2-1	4.0 ± 0.6^c^	3.1 ∗ 10^9^	47.64 ± 0.6^d^	1.89 ± 0.23^e^
GA0.2-2^a^	4.7 ± 0.5^c^	2.1 ∗ 10^9^	48.17 ± 1.9^d^	2.25 ± 0.25^e^
GA0.2-3	4.1 ± 0.2^c^	5.0 ∗ 10^9^	49.72 ± 2.7^d^	2.04 ± 0.16^e^
GA0.2-4^a^	3.8 ± 0.4^c^	3.4 ∗ 10^9^	55.26 ± 1.3^d^	2.12 ± 0.17^e^
GA0.2-5	3.0 ± 0.4	2.3 ∗ 10^9^	46.63 ± 1.5^d^	1.40 ± 0.61^e^
GA0.2-6	4.2 ± 0.3^c^	2.2 ∗ 10^9^	43.05 ± 1.1	1.81 ± 0.09^e^
GA0.2-7	4.8 ± 0.3^c^	9.6 ∗ 10^9^	38.82 ± 2.7	1.86 ± 0.10^e^
GA0.2-8^a^	4.7 ± 0.3^c^	9.0 ∗ 10^9^	52.16 ± 2.6^d^	2.43 ± 0.20^e^
GA0.4-1	2.4 ± 0.2	2.0 ∗ 10^9^	42.81 ± 2.4	1.03 ± 0.12^e^
GA0.4-2	3.8 ± 0.3^c^	2.3 ∗ 10^9^	42.75 ± 1.9	1.61 ± 0.13^e^
GA0.4-3^a^	3.3 ± 0.8	3.1 ∗ 10^9^	45.97 ± 2.0^d^	1.52 ± 0.25^e^
AEC0.2-1^a^	4.7 ± 0.2^c^	7.4 ∗ 10^9^	52.22 ± 2.8^d^	2.47 ± 0.11^e^

^a^Samples that were selected for the HPLC analysis.

^b^The biomass contained spores that did not settle down after centrifugation, therefore affecting and showing higher biomass yield than the other samples.

^c^,^d^,^e^Sample results were statistically significantly different from WT.

**Table 2:** Results of *B. subtilis* and its mutant GA inhibitor selected candidates as AA g/(100 g_sample_).

AA	WT	WT2	GA0.05-2	GA0.05-7	GA0.05-8	GA0.01-1	GA0.01-4	GA0.01-6	GA0.1-1	GA0.2-2	GA0.2-4	GA0.2-8	GA0.4-3
Asp ac	4.62	4.33	5.16	5.3	4.72	6.05	5.09	4.84	6.32	5.23	5.56	6.09	5.93
Glu ac	8.92	8.4	10.1	11.7	6.97	7.52	9.57	9.79	7.75	9.51	9.89	7.97	9.4
Ser	1.28	1.15	1.72	1.28	1.79	1.73	1.61	1.7	2.11	1.77	1.72	1.99	1.42
His	0.81	0.82	1.17	1.23	1.01	1.06	1.17	1.1	1.17	1.29	1.33	1.42	1.23
Gly	1.99	2.03	2.25	2.55	2.3	3.07	2.29	2.24	3.04	2.34	2.42	2.48	2.38
Thr	1.65	1.58	2.09	1.86	2.17	2.26	1.95	2	2.55	1.99	2.12	2.38	1.93
Arg	2.04	1.94	2.58	2.53	2.9	3.03	2.62	2.57	3.56	2.9	2.76	2.84	2.55
Ala	4.29	4.23	4.53	4.49	4.62	3.95	4.46	4.36	4.25	4.6	4.71	4.32	5.23
Tyr	1.97	1.98	2.24	2.1	1.72	1.92	2.2	2.25	2.03	2.26	2.35	2.18	2.14
Cys	0.16	0.17	0.5	0.03	0	0.06	0	0.62	0	0	0	0	0
Val	2.58	2.63	2.8	2.98	2.76	3.52	2.63	2.7	3.61	3.09	3.12	3.15	3.19
Met	1.05	0.78	1.34	1.3	1.03	1.38	1.19	1.28	1.5	1.15	1.38	1.45	1.34
Phe	1.82	1.83	2.25	2.29	1.93	2.13	2.1	2.11	2.27	2.18	2.34	2.52	2.1
Ile	2.27	2.31	2.63	2.95	2.03	2.67	2.52	2.54	2.72	2.56	3.15	3.13	3.24
Leu	3.24	3.27	3.87	4.04	3.76	4.26	3.65	3.65	4.54	3.69	4.05	4.4	3.9
Lys	3.09	3.06	3.38	3.48	2.18	3.21	3.14	3.3	3.43	3.28	3.71	3.96	3.59
Pro	1.95	1.84	2.38	2.35	2.46	2.5	1.97	2.24	2.37	1.87	2.04	2.2	1.94
Protein content, %	43.7	42.4	51.0	52.5	44.4	50.3	48.2	49.3	53.2	49.7	52.6	52.5	51.5

**Table 3:** Results of *B. subtilis* and its mutant MSO and AEC inhibitor selected candidates as A g/(100 g_sample_).

AA	WT	MSO0.01-1	MSO0.01-6	MSO0.025-1	MSO0.025-2	MSO0.025-3	MSO0.025-5	MSO0.05-1	AEC0.2-1
Asp ac	4.62	6.27	4.98	5.2	5.63	6.04	5.77	6.12	6.09
Glu ac	8.92	7.77	9.25	10.27	12.61	11.31	10.06	7.82	8.99
Ser	1.28	1.64	1.8	1.4	1.15	1.86	1.86	2.12	1.86
His	0.81	1.15	1.2	1.24	1.26	1.33	1.19	1.18	1.41
Gly	1.99	3.28	2.36	2.61	2.57	2.56	2.4	3	2.57
Thr	1.65	2.26	1.92	2	1.87	2.36	2.24	2.49	2.24
Arg	2.04	3.32	2.74	2.45	2.38	2.65	2.62	3.51	2.89
Ala	4.29	4.15	4.49	4.55	4.52	4.66	4.89	4.22	4.94
Tyr	1.97	1.95	2.41	2.18	2.19	2.3	2.06	1.94	2.59
Cys	0.16	0.04	0	0.43	0	0	0.35	0.58	0
Val	2.58	3.5	2.57	3.22	3.16	3.21	3	3.36	3.31
Met	1.05	1.41	1.07	1.38	1.38	1.26	1.27	1.37	1.3
Phe	1.82	2.25	2.11	2.25	2.21	2.32	2.2	2.24	2.33
Ile	2.27	2.79	2.44	3.07	3.05	3.02	2.95	2.7	3.34
Leu	3.24	4.47	3.53	4.17	4.17	4.44	4.23	4.51	4.44
Lys	3.09	3.19	3.12	3.7	3.73	3.96	3.03	3.28	3.88
Pro	1.95	2.67	1.91	2.13	2.16	2.32	2.31	2.52	2.52
Protein content, %	43.7	52.1	47.9	52.2	54.0	55.6	52.4	53.0	54.7

**Table 4:** AAS (%) for each AA by referencing fish meal.

Sample	His	Thr	Arg	Val	Met	Phe	Ile	Leu	Lys	The first-limiting AA
WT	76.9^a^	86.4	81.9	108.7	79.6	352.9	126.1	156.4	91.4	His
GA0.01-1	87.4	102.8	105.7	128.9	90.8	358.9	128.8	178.7	82.5^a^	Lys
GA0.01-4	100.7	92.6	95.4	100.5	81.8^a^	369.2	126.9	159.8	84.2	Met
GA0.01-6	92.6	92.8	91.5	100.9	86.0^a^	362.7	125.1	156.2	86.5	Met
GA0.05-2	95.2	93.8	88.8	101.1	87.0	373.9	125.2	160.1	85.6^a^	Lys
GA0.05-7	97.2	81.1^a^	84.5	104.5	82.0	369.7	136.4	162.3	85.6	Thr
GA0.05-8	94.4	111.8	114.6	114.5	76.8	368.4	111.0	178.7	63.4^a^	Lys
GA0.1-1	91.3	109.7	117.4	125.0	93.4	361.6	124.1	180.0	83.3^a^	Lys
GA0.1-1	102.4	110.4	121.2	135.3	80.5^a^	363.6	117.6	181.4	83.8	Met
GA0.2-2	107.7	91.6	102.4	114.5	76.6^a^	371.7	125.0	156.6	85.3	Met
GA0.2-4	104.9	92.2	92.1	109.2	86.9^a^	377.0	145.4	162.4	91.1	Met
GA0.2-8	112.2	103.7	94.9	110.5	91.5^a^	406.8	144.7	176.8	97.5	Met
GA0.4-3	99.1	85.8^a^	86.9	114.1	86.2	345.6	152.7	159.8	90.1	Thr
MSO0.01-1	91.6	99.3	111.8	123.7	89.6	366.0	130.0	181.0	79.1^a^	Lys
MSO0.01-1	113.7	89.5	88.8	129.2	80.9^a^	357.9	139.8	169.4	89.5	Met
MSO0.01-6	104.0	91.7	100.4	98.8	74.0^a^	373.3	123.6	155.5	84.2	Met
MSO0.025-1	98.6	87.7	82.3^a^	113.6	87.5	365.3	142.7	168.5	91.6	Arg
MSO0.025-2	96.8	79.2	77.3^a^	107.8	84.6	346.8	137.1	162.9	89.2	Arg
MSO0.025-3	99.3	97.1	83.6	106.3	75.0^a^	353.6	131.8	168.5	92.0	Met
MSO0.025-5	94.2	97.8	87.7	105.4	80.3	355.8	136.6	170.3	74.7^a^	Lys
MSO0.05-1	92.4	107.5	116.2	116.8	85.6	358.2	123.6	179.5	80.0^a^	Lys
AEC0.2-1	107.0	93.7	92.7	111.4	78.7^a^	361.0	148.2	171.2	91.6	Met

^a^Highlighted results for the AAS scores corresponding to the first limiting AA of the respective sample.

## Data Availability

The data that support the findings of this study are available from the corresponding author upon reasonable request.
